# Surface-Engineered Nanocontainers Based on Molecular Self-Assembly and Their Release of Methenamine

**DOI:** 10.3390/polym10020163

**Published:** 2018-02-08

**Authors:** Minghui Zhang, Jinben Wang, Pei Zhang, Haike Yan

**Affiliations:** 1 Beijing National Laboratory for Molecular Sciences, Key Laboratory of Colloid, Interface and Chemical Thermodynamics, Institute of Chemistry, Chinese Academy of Sciences, Beijing 100190, China; zhangminghui@iccas.ac.cn (M.Z.); hkyan@iccas.ac.cn (H.Y.); 2 University of Chinese Academy of Sciences, Beijing 100049, China

**Keywords:** halloysite nanotubes, polymerizable geminized monomer, surface engineer, molecular self-assembly, nanocomposite, controlled release, cross-linking agent

## Abstract

The mixing of polymers and nanoparticles is opening pathways for engineering flexible composites that exhibit advantageous functional properties. To fabricate controllable assembling nanocomposites for efficiently encapsulating methenamine and releasing them on demand, we functionalized the surface of natural halloysite nanotubes (HNTs) selectively with polymerizable gemini surfactant which has peculiar aggregation behavior, aiming at endowing the nanomaterials with self-assembly and stimulative responsiveness characteristics. The micromorphology, grafted components and functional groups were identified using transmission electron microscopy (TEM), thermogravimetric analysis (TGA), Fourier transform infrared (FTIR) spectroscopy, and X-ray photoelectron spectroscopy (XPS). The created nanocomposites presented various characteristics of methenamine release with differences in the surface composition. It is particularly worth mentioning that the controlled release was more efficient with the increase of geminized monomer proportion, which is reasonably attributed to the fact that the amphiphilic geminized moieties with positive charge and obvious hydrophobic interactions interact with the outer and inner surface in different ways through fabricating polymeric shell as release stoppers at nanotube ends and forming polymer brush into the nanotube lumen for guest immobilization. Meanwhile, the nanocomposites present temperature and salinity responsive characteristics for the release of methenamine. The combination of HNTs with conjugated functional polymers will open pathways for engineering flexible composites which are promising for application in controlled release fields.

## 1. Introduction

Halloysite nanotubes (HNTs) are types of naturally occurring 1:1 clays with nanotubular structures, possessing a coil structure with external diameters of 50~80 nm, size of lumen 10~15 nm, and morphological similarity to multiwalled CNTs [1,2,3]. In halloysite nanotubes, the outer portions are primary siloxane groups (Si−O−Si), and the gibbsite octahedral sheet (Al−OH) groups are exposed in inner lumen surface. However, most of the hydroxyl groups are inner groups because of the multi-layer structure, resulting in the low density of hydroxyl groups on the outer surface. The peculiarity determines a negatively charged outer surface and a positively charged lumen in the range of pH 2~8 [4,5,6]. Due to the unique combination of a tubular nanostructure capable of being loaded with chemically active agents and rich functionality, HNTs have regained attention in materials science as nanocontainers for controlled release of different chemical agents including corrosion inhibitors, biocides, antifouling agents, drugs, etc. [7,8,9,10,11,12].

Developing nanocontainers which have the ability to encapsulate desired active materials efficiently, and maintain them successfully in the inner cavity during long time periods, is the essential and critical approach in establishing new encapsulation system. Beyond that, a desired property of the envisaged containers is the immediate or prolonged release of encapsulated active material triggered by specific changes in the environment surrounding the container or directly in the container shell [13]. HNTs can offer many benefits for introducing in polymer composites due to their characteristics of tubular nanostructure, high stability, excellent biocompatibility, low cost, and proper mechanical properties, which may replace the more expensive CNTs in designing of smart composite materials and multi-functional nanocomposites [14,15,16,17,18]. However, how to functionalize a natural halloysite nanotube—to make it responsive to environmental conditions such as pH, temperature, and ionic strength—is being explored very little. Furthermore, it is critical that achieving optimal structure and properties of HNTs-based nanocomposites through controlling dispersion and spatial arrangement of HNTs within the nanocomposite. The combination of polymers and nanoparticles can also exhibit excellent electrical, optical, or mechanical properties [19]. Covalently attaching functional polymer chains onto the surface of nanotubes has recently attracted considerable attention because it endows the surfaces with controllable architecture and features, which may provide opportunities for the preparation of environmentally responsive hybrid materials [20]. Via “grafting to” method, many different polymer chains could be conjugated onto the surface of nanotubes, which will be an interesting direction for modifying halloysite nanotubes as smart nanocarrier [21,22].

**Scheme 1:**
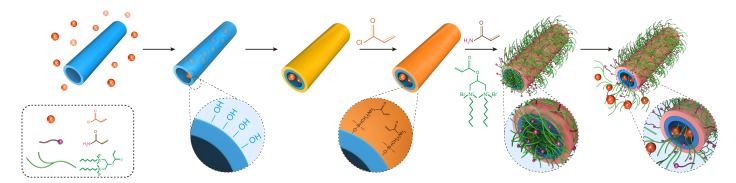
Schematic illustration of the salt-responsive nanocontainers based on HNTs encapsulated in an amphiphilic copolymer layer.

Amphiphilic polymers are effective colloidal stabilizers of aqueous dispersions due to their strong inter-molecular interaction [23]. In the case of amphiphilic polyelectrolyte, the electrostatic and intra/inter-molecular interactions between hydrophobic moieties provide coordination to the formation of adsorption layers, especially for the surface with opposite charge. In view of the substantial interactions between inorganic colloid particles and geminized amphiphilic polyelectrolyte (PAGC_8_), which has ultrahigh charge density and strong hydrophobic interaction, the same structure with a more hydrophobic group was assumed to behave remarkably in the physico-chemical engineering for flexible surface composites [24]. Acrylamide (AM) is the most important amide-containing acrylic monomers with high polymerization activity. The related polyacrylamide polymers are comparatively low-cost and water-soluble polymers with a unique combination of properties. The copolymerization of AM and AGC can form more viscoelastic shell on the surface of HNTs with fence-like structure, in which the properties of shell will be modulated by the external stimuli such as temperature and salinity. In this work, we modified the surface of HNTs with acryloyl group, by in situ polymerization of AGC_12_ monomer with acrylamide using a one-pot random copolymerization technique to create novel nanocomposites, aiming at fabricating controllable assembling nanocomposite materials for efficiently encapsulating active guest molecules of methenamine and releasing with stimulative responsibility (abbreviated as AGC_12_ with the structure as shown in Scheme 1). As an important chemical product, methenamine has been applied in many fields. Such as antiseptics, pesticides, cross-linking agents, catalytic agents, and so on. The use of a stimuli-responsive nanocontainer can improve the use efficiency of methenamine significantly.

## 2. Materials and Methods 

### 2.1. Materials

**Scheme 2:**
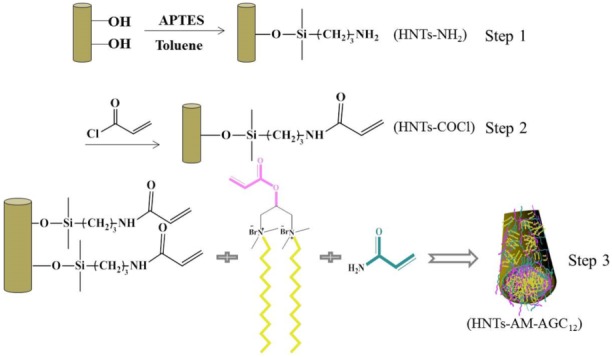
Synthetic routes for polymerizable gemini surfactants.

The amphiphilic monomer AGC_12_ was prepared and purified according to our previous work, and their synthetic routes are shown in Scheme 2 [25]. The detailed synthetic procedure of AGC_12_ was given in Supplementary Materials. Deuterium oxide (99.9%) and chloroform-d (99.9%) were purchased from Acros (Geel, Belgium. Acryloyl chloride, 1-bromine octane and solvents were purchased from Beijing Chemical Co. (analytical reagent (AR) grade, Beijing, China). The used halloysite clay nanotubes (HNTs) were obtained from Nanyang Hongfa Bentonite Company in Nanyang, China. Acrylamide (AM) was obtained from Acros and purified via recrystallization before use. 3-aminopropyltriethoxysilane (APTES, 98%), serving as a silica source was supplied by Sigma-Aldrich (St Louis, MI, USA). and used as received. Methenamine, used for guest loading, was purchased from Alfa Aesar (Ward Hill, MA, USA).

### 2.2. Sample Preparation

**Scheme 3:**
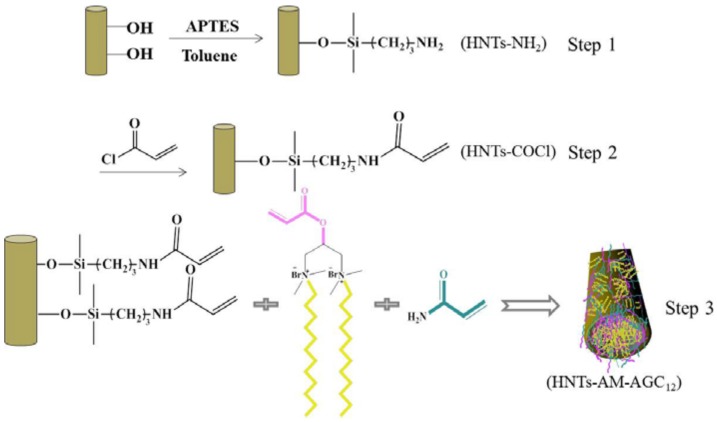
General strategy for surface functionalization of halloysite nanotubes (HNTs). (The modified halloysite nanotubes were abbreviated as HNTs-NH_2_ in step 1, HNTs-COCl in step 2, and HNTs-AM-AGC_12_ in step 3 respectively).

The modification of halloysite nanotubes was carried out through random copolymerization of the amphiphilic monomer with the acryloyl groups on halloysite nanotube surface. Details on the synthesis of grafted halloysite nanotubes and amphiphilic monomer are given in the Supplementary Materials. The samples “HNTs-AM-AGC”, “HNTs-AM-53%AGC” and “HNTs-53%AM-AGC” were prepared according to the feeding mass ratio “1:1:1, 1:1:0.53 and 1:0.53:1” respectively. The in situ synthesis procedure of halloysite nanocomposites was carried out via random copolymerization initiated by an initiator V50, by which the amphiphilic units were grafted onto the surface of halloysite. A schematic of the synthesized procedure is shown in Scheme 3.

### 2.3. Characterization

The structure, amount of grafted groups, and micromorphology of the surface-engineered HNTs were characterized using FTIR, XRD, XPS, TGA, and TEM methods.

Fourier transform infrared (FTIR) spectroscopy: FTIR spectra in KBr were determined at room temperature in the spectral region 400−4000 cm^−1^ by means of an FTIR spectrophotometer (Bruker Tensor-27, Billerica, MA, USA). An average of 30 scans per sample using a nominal resolution of 4 cm^−1^ was registered.

X-ray diffraction (XRD) analysis: The crystal structures of the HNTs, modified HNTs were determined by a Rigaku D/max-3A diffractometer (Tokyo, Japan) using a Cu Kα-ray line at a scan rate of 8°/min.

X-ray photoelectron spectroscopy (XPS): X-ray photoelectron spectroscopy (XPS) was taken with a Thermo Scientific ESCALab 250Xi (Waltham, MA, USA), using 200 W monochromatic X-ray source (Al Kα). The 500 μm X-ray spot and the base pressure 3 × 10^−10^ mbar of analysis chamber were applied. The pass energy was set at 284.8 eV for the hydrocarbon C1s line from adventitious carbon. 

Thermogravimetric analysis (TGA): Thermogravimetric analysis was conducted using a thermogravimetric analyzer TGA (TG 209 NETZSCH, Selb, Germany). The samples were heated under nitrogen atmosphere from room temperature to 800 °C at a heating rate of 10 °C/min.

Transmission electron microscopy (TEM): The nanotubular shapes of HNTs were observed with a Philips Tecnai 10 TEM (Amsterdam, the Netherlands) at 200 kV acceleration voltage, HNTs-AM, HNTs-GC_12_, and HNTs-AM-GC_12_. The samples were ground into powders and dispersed in water via ultrasonication. Then a drop of the solution with a concentration of about 0.1% was dripped onto carbon coated copper grids and dried at 35 °C. The final grids were used for TEM observation.

### 2.4. Nanotube Loading Procedure

To entrap methenamine, grafted halloysite (HNTs-COCl) was mixed as a dry powder with a saturated solution of methenamine in ethanol (1.2 g/mL). A beaker containing methenamine and HNTs-COCl suspension was transferred to a vacuum jar, which was then evacuated using a vacuum pump. The suspension was vacuumed for 1~2 h three times, and this process was repeated three times in order to increase loading efficiency. Subsequently, the nanotubes were washed with distilled water accompanied with centrifugation separation and then polymerized with amphiphilic monomer in a “one-pot” mode, obtaining the loaded nanocomposites.

### 2.5. Functional Chemicals Release Performance of Surface Engineered HNTs

Methenamine as a crosslinking agent is capable of crosslinking monomers or polymers to form gel. However, the release rate of the crosslinking agent is a crucial factor in the process of gelation. The efficiency of filling crosslinking agent into halloysite tubes and its release were investigated by concentration measurements via a UV–Visible spectrophotometer. 0.8 g HNTs-AM-AGC_12_ nanocomposite was put into a dialysis tube (MWCO = 2500), and dialyzed against 123.2 mL distilled water at room temperature. 2 mL solution was taken out from dialysis solution at set intervals to measure the concentration of the released crosslinking agent. At the same time, 2 mL distilled water was added to the mixed solution to keep the volume constant. 

**Figure 1:**
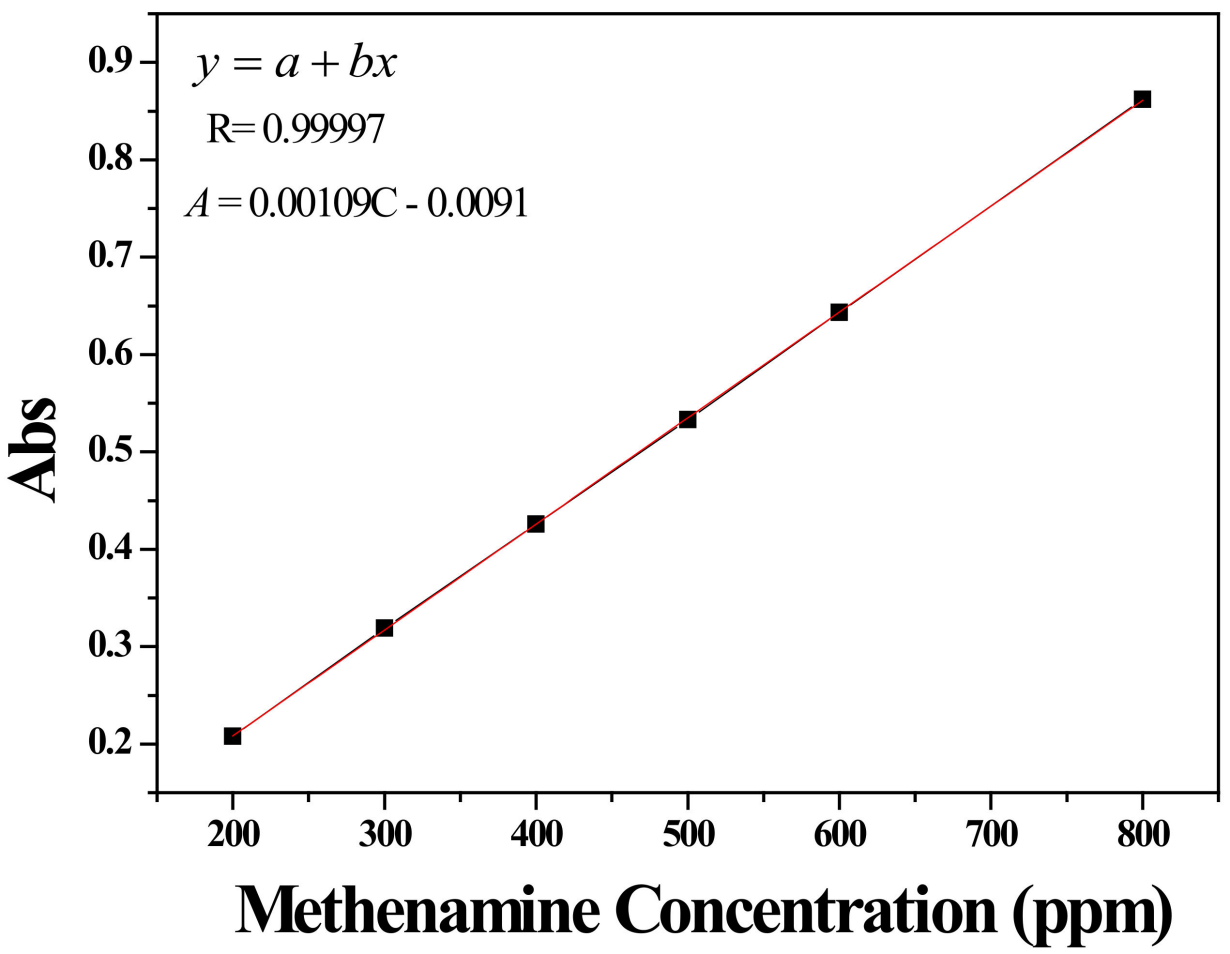
The calibration absorption curve of methenamine. The cumulative release concentration (*C*) at a particular time (t) can be calculated according to the equation.

The amount of methenamine released from nanocomposites was determined according to a standard curve measured by UV absorbance at 215.5 nm, as shown in Figure 1.

### 2.6. Change of Particle Size with Methenamine Release Dynamics

To explore the factors of controlled release kinetics, measurement of the average particle size of HNTs-AM-AGC_12_ nanocomposites was performed using a Zetasizer Nano ZS (Malvern Instruments, Malvern, UK). The same concentration of nanocomposites and corresponding condition were chosen referring to methenamine release process. The suspension was measured rapidly with three times repeat to obtain the tendency of particle size with release time.

## 3. Results and Discussion

The HNTs bearing random AGC_12_ units were prepared according to the synthetic route shown in Scheme 1. The characterization was aimed at proving and highlighting the structure besides checking the release features in water and their environmental responsibility.

### 3.1. Synthesis and Characterization of Modified HNTs

**Figure 2:**
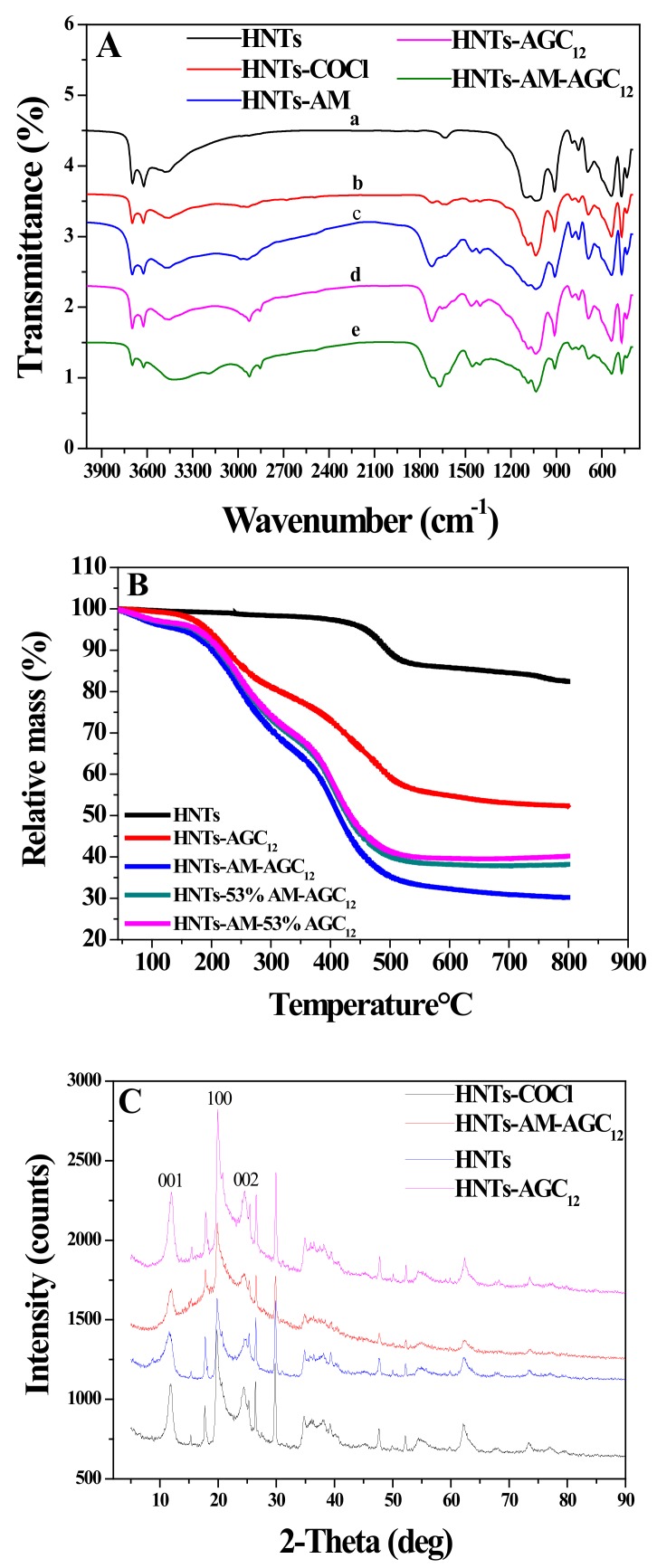
(**A**) FTIR spectra of (a) pristine HNTs, (b) HNTs–COCl, (c) HNTs–AM, (d) HNTs–AGC_12_ and (e) HNTs-AM-AGC_12_ nanocomposites; (**B**) TGA curves of pristine HNTs (HNTs, 1), HNTs-AGC_12_, HNTs grafted with AGC_12_ (2), HNTs grafted with AGC_12_ and AM under N_2_ (3); (**C**) XRD spectra of halloysite before and after modification.

Figure 2A shows the typical FTIR spectra of HNTs before and after modification with different groups. The distinct peaks at 3703 and 3625 cm^−1^ can be attributed to the stretching vibrations of inner and surface –OH groups. Compared with the pristine HNTs, new peaks were exhibited in the range of 3100~1200 cm^−1^, after substituting the hydroxyl groups with –CONH_2_ and alkyl chain groups at the surface of HNTs, and the IR peak of surface alcohol from 3200 to 3500 disappeared. The presence of alkyl chain in grafted HNTs with AGC_12_ was demonstrated by the –CH_3_ and –CH_2_– stretching vibration bands at 2960, 2870 cm^−1^ and 2925, 2850 cm^−1^, respectively. Furthermore, carbonyl vibrations appeared at 1665 and 1724 cm^−1^ for the amide and carboxylic ester groups.

The grafting degree can be estimated from TGA curves of original HNTs and modified HNTs respectively, as shown in Figure 2B. The pristine HNTs showed excellent thermal stability below 800 °C, for which, the slight mass loss before 200 °C can be attributed to the adsorbed water existing in the surface, cavity, or interlayers of HNTs. The distinct mass loss range from 200 to 800 °C was relating to the degradation of the organic fractions, and the grafting degree was evaluated by comparing the residual masses, of which the values are 30, 38, 40, and 52% for HNTs-AGC_12_, HNTs-AM-53%AGC_12_, HNTs-53%AM-AGC_12_, and HNTs-AM-AGC_12_, respectively. To further illustrate the effect of the incorporation of the different kinds of HNTs on the crystal properties of the nanocomposites, XRD experiment was carried out and the X-ray diffraction patterns of halloysite before and after modification were shown in Figure 2C. The diffraction peak (001) corresponding to a multilayer wall spacing of 0.74 nm, identifies the ‒7 A˚ characteristic of halloysite. For halloysite without amphiphilic component modifying, sharp and well-resolved basal reflection lines showed an ordered arrangement of silico-aluminate, such as peaks of (001), (100), and (002). However, gradual changes in number and resolution of the characteristic peaks were observed after modifying by amphiphilic monomer, which indicates differences in crystallinity and partly grafted to the inner surface.

**Figure 3:**
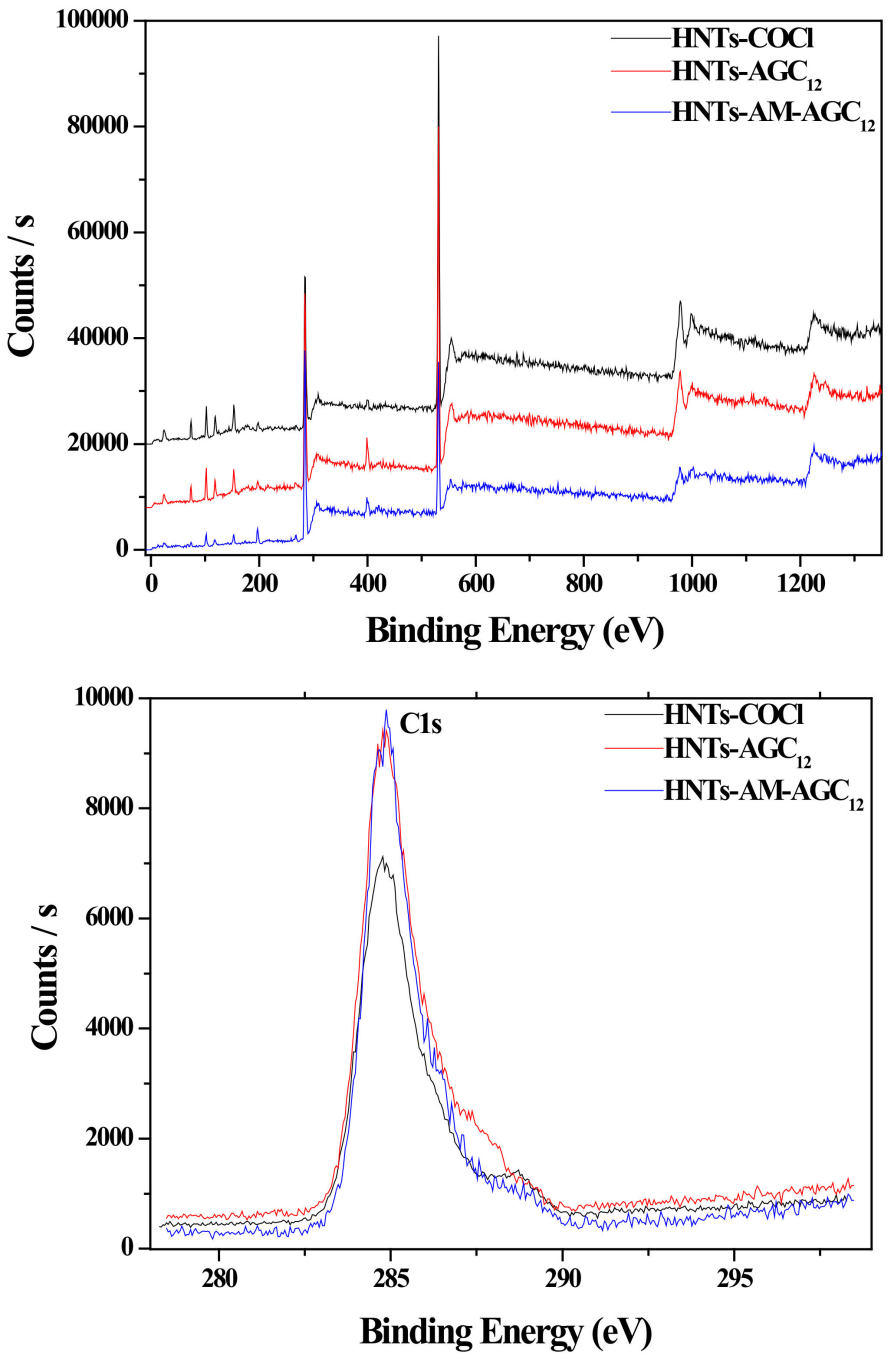

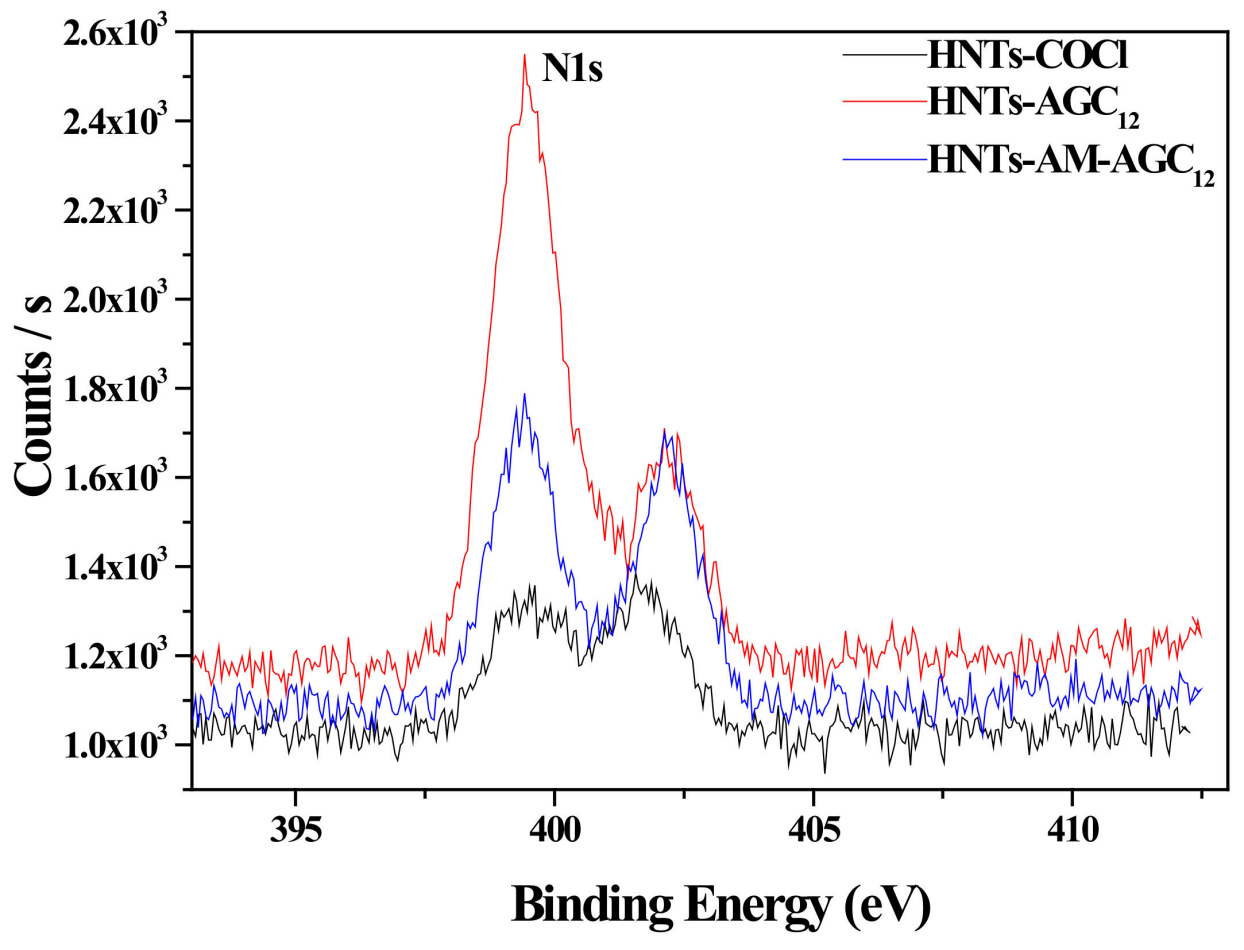
XPS spectra of HNTs before and after modification.

The surface composition of nanotubes was determined by XPS analysis, as shown in Figure 3. The XPS spectra of modified HNTs confirmed the presence of aluminum (Al2s and Al2p) and silicon (Si3p), in accordance with the composition of original HNTs reported elsewhere [26]. Both of N1s and C1s peaks can be observed after grafting with AGC_12_ and acrylamide onto the HNTs. In addition, the peak of N1s presented stronger signal in the case of HNTs-AGC_12_ than that of HNTs-AM-AGC_12_, suggesting a high grafting degree of acrylamide monomer. The peak at 284.5 eV in the spectrum of modified HNTs corresponds to the C=C and C–C bonds, of which the peak areas appear to be larger as a result of the increased amount of organic contents grafted on the HNTs, successively.

**Figure 4:**
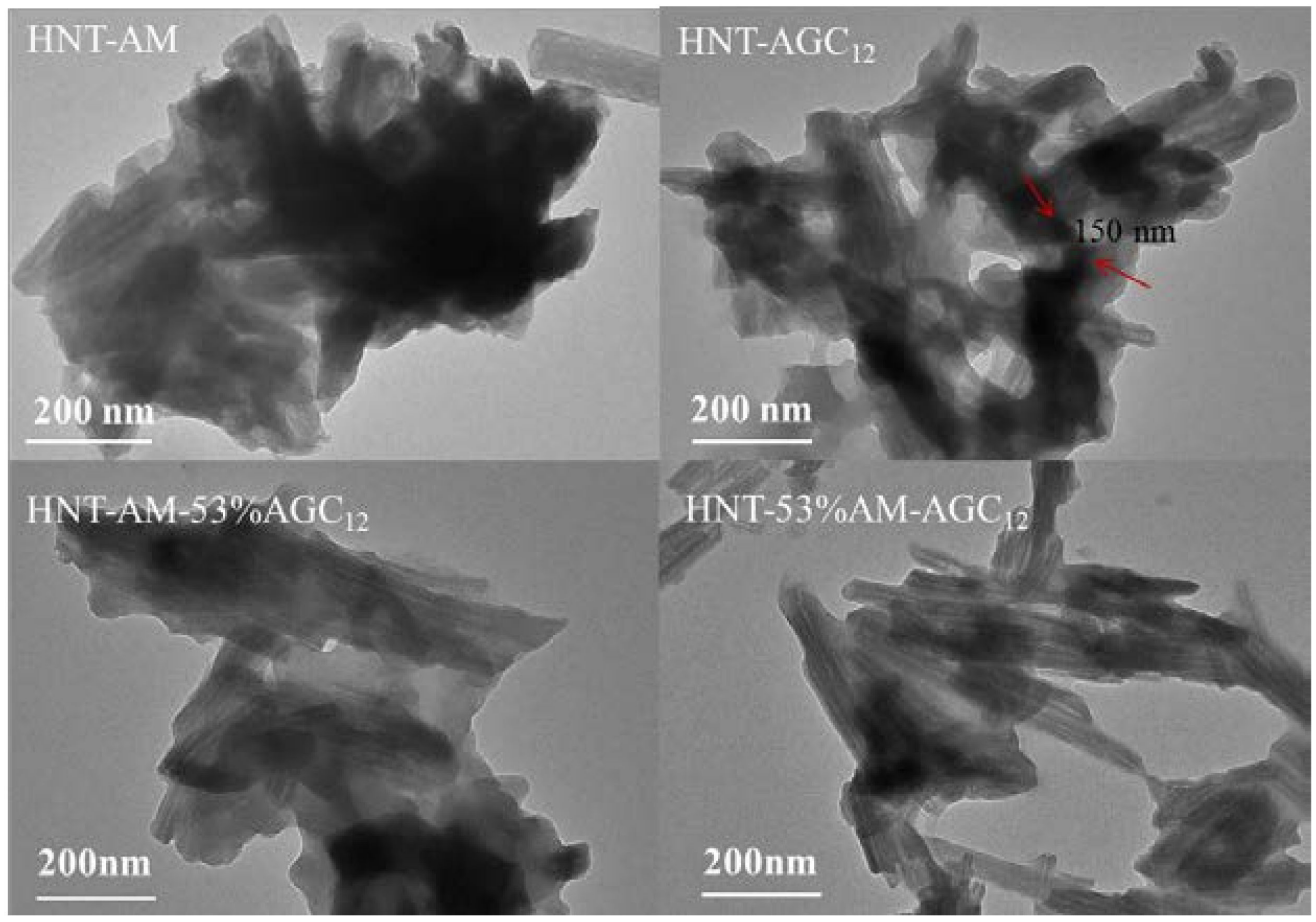
TEM images of grafted HNTs with different constituents.

TEM micrograph, as shown in Figure 4, confirms that substantial changes occur in the morphology after grafting AGC_12_ and acrylamide monomer onto the surface of HNTs and that the surface of HNTs-AGC_12_ and HNTs-AM-AGC_12_ exhibit various microstructures in the case of modification with different polymers.

It can be also observed that the surface-engineered HNTs were entangled and stacked with each other in various ways, and a regular continuous network was formed at appropriate ratio of AGC_12_ and AM, which indicates self-assembly behavior on the HNTs surface. The average diameters of the nanocomposites are proximately in the range of 500 ± 2000 nm, however, the surface-engineered HNTs with polyacrylamide stacked tightly, in which the HNTs arranged on an individual level. In the case of introducing amphiphilic AGC_12_, the spatial distribution of HNTs in nanocomposites was controlled through the polymer brush such as the image of HNTs-AGC_12_.

### 3.2. Taking Surface-Engineered HNTs as Carriers for Methenamine Release

The hollow lumen of the halloysite nanotubes with an inner diameter of 15~20 nm has good loading capacity. Nevertheless, the size is not suitable for application in controlling release. The release of active substances incorporated into nanocomposites depends on the surface shell grafted on the HNTs: the shape, porosity, and thickness of the layers. The release performances of all nanocontainers with successfully encapsulated methenamine were studied in aqueous solutions at a neutral pH. In addition, the influences of temperature and salt on the release kinetics were investigated to explore the optimal conditions at which the release characteristic possesses surrounding media responsiveness.

**Figure 5:**
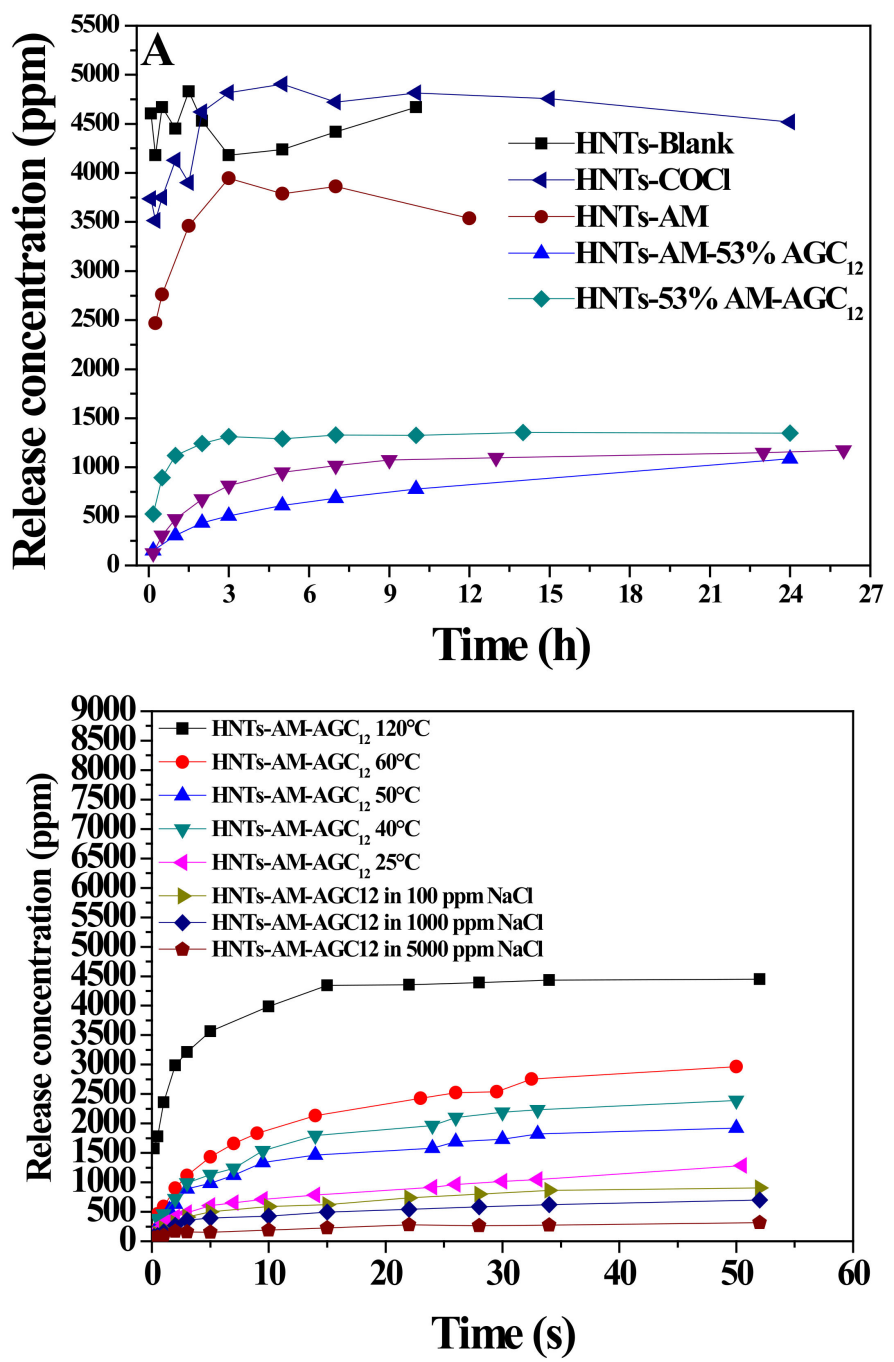
The release of methenamine during the aging of nanocontainers at different surrounding media.

**Figure 6:**
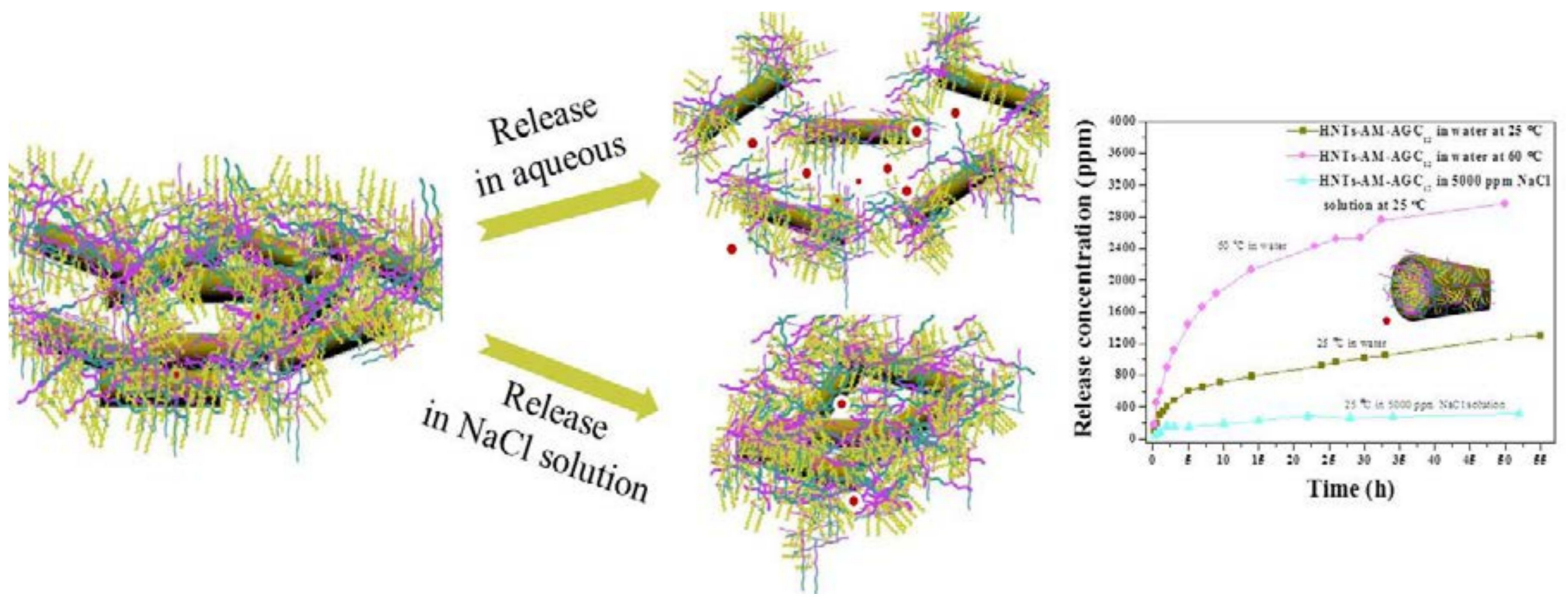
Scheme of methenamine release from nanocomposite at different conditions.

It was found that the acrylated HNTs (HNTs-COCl) had a load capacity similar to blank HNTs, and the created nanocomposites grafted with different components had various properties with respect to the controlled release of methenamine. Specifically, nanocontainers without amphiphilic components (HNTs-COCl, HNTs-AM) embodied an obvious phenomenon of sudden release, lacking controlled-release potential, as shown in Figure 5A. By comparison, the modified HNTs with amphiphilic AGC_12_ has excellent controllable drug release performance, by which the release rate can be adjusted flexibly depending on the graft condition; the structure of grafted layers; and the medium properties such as pH, temperature, salinity, etc. As seen in Figure 5B, the nanocomposites behave temperature and salinity responsive characteristics in the release of methenamine, for which, the high release rate was presented in the condition of higher temperatures and delayed release took place in the circumstance of higher NaCl concentrations. Thus, modification of halloysite nanotubes with amphiphilic monomer is a feasible way to achieve controlled release of cross-linking agent, as illustrated in Figure 6. Moreover, it is needed to further study the nature of release characteristics in different environments. 

### 3.3. Particle Size Variations of the Nanocarriers in the Releasing Process under Various Conditions

Usually, the release rate of guest molecules incorporated into carrier essentially depends on the following factors: the specific host–guest interactions, the porosity and size of the host container, as well as the shape of the dosage form [27]. As an introduction of amphiphilic monomer with high charge density into the nanocomposites, the interaction of copolymer with halloysite is significantly stronger because they have more charged sites per single molecule. In addition, the halloysite lumen with amphiphilic molecules grafted on it may behave like a sponge for physisorption, increasing the adsorption capacity and fixing the guest molecules. Thus, the nanocomposite has swelling properties in water due to the interaction of chain–chain and chain–surface between copolymer and HNTs, and the release of methenamine depends on the swelling and disaggregation of the copolymer.

**Figure 7:**
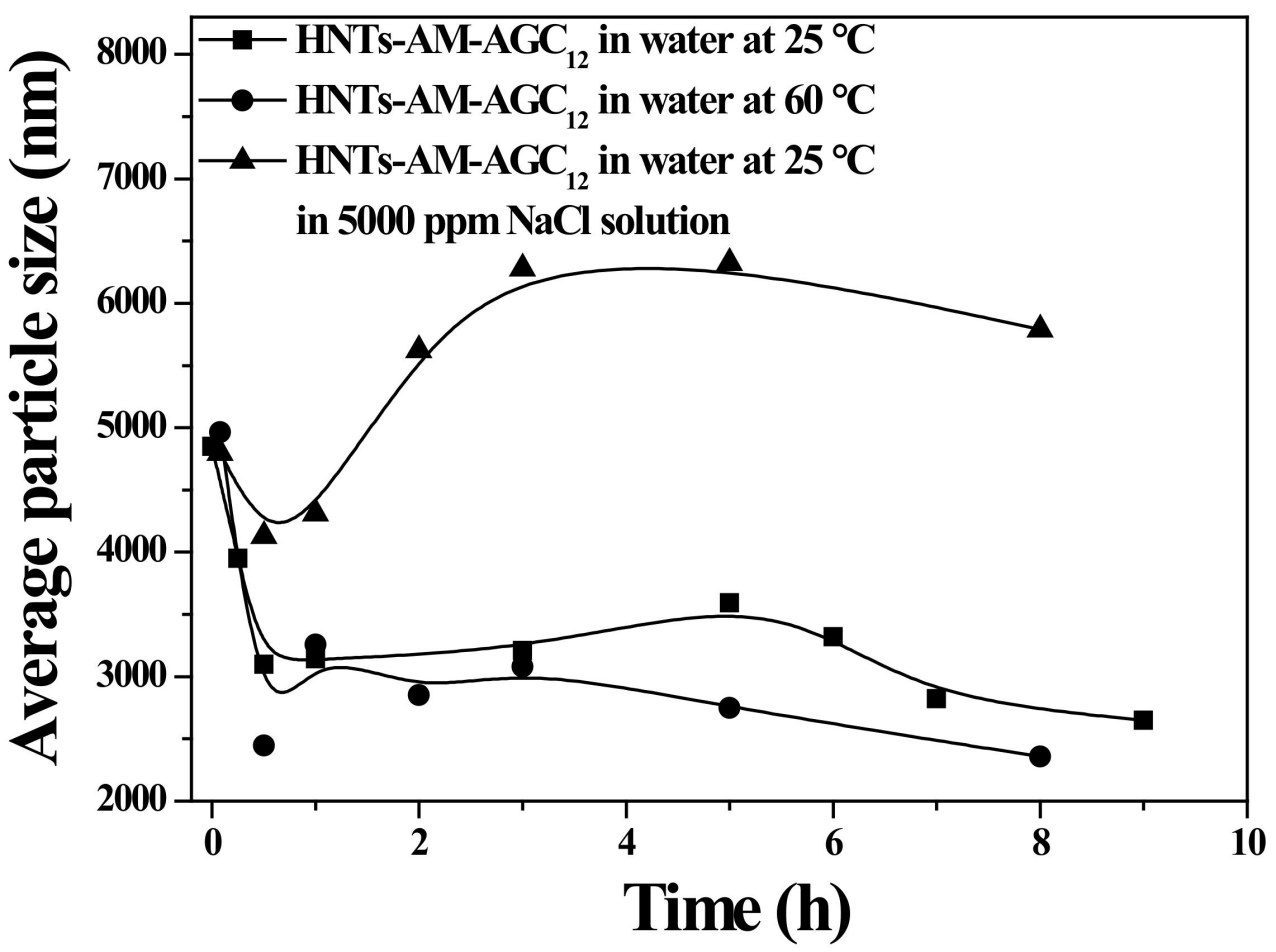
The particle size change during the aging of nanocontainers in water at different environments.

As we know, increases in temperature can enhance the flexibility of side chain, resulting in an increase in the swelling degree of the nanocontainers. As shown in Figure 7, the particle size of nanocontainers decreased with the releasing time in aqueous solution at 25 °C and decreased further as the temperature increased up to 60 °C; a tendency which corresponds to the kinetics of methenamine release. The enhancement of ionic strength is capable of weakening the electrostatic interactions of cationic amphiphilic molecules, transforming the aggregation and increasing the adsorbing capacity. On that basis, the interactions of copolymer in the nanocontainers including chain–chain hydrophobic interaction, electrostatic interaction between backbone and HNT surface, were adjusted to form a more tight structure. Meanwhile, the permeation ability of water from the outside into the nanotube is also restrained, which was the reason of that the particle size increases during the aging of nanocontainers in NaCl solution. It is reasonable to speculate that the disaggregation of nanoparticles leads to the release of methenamine in part and the release rate continues to increase with time at the condition of unchanged particle size. The release kinetics can be explained as the following steps: the collapsing of entangled modified HNTs and the further diffusion of guest molecules from the modified layers and the lumen of modified HNTs. Interaction among particles based on amphiphilic constituents makes the modified HNTs entangle together, resulting in opening the blocked tube partially. In this way, the release kinetics is from the dissolution of nanoparticles. Furthermore, the release process without particle size change may be ascribed to the formation of polymer brush into the nanotube lumen grafting by amphiphilic monomer in the inner surface.

## 5. Conclusions

Hybrid nanoparticles with tunable properties were prepared by selectively anchoring cationic amphiphilic polyelectrolytes at the outer surface of halloysite. Through different innermost/outermost surface modification of clay nanotubes, a functional nanocomposite system was constructed which has a large encapsulating capacity for a cross-linking agent (methenamine) and ideal controllable release behavior under the stimulation of environmental factors such as temperature and salinity. The conducted studies have revealed that the loading capacity and release behaviors of the hybrid nanocarriers were also dependent on its surface chemical composition such as the loading efficiency being greatly increased and the release rate becoming controllable with the increase of cationic geminized monomer AGC_12_ grafted at the HNTs surface. In virtue of the positive charge and obvious hydrophobic interactions, the outer and inner HNTs surfaces were engineered by polymeric shell forming by block copolymer poly(acrylamide-*b*-AGC_12_) supporting as release stoppers at nanotube ends. However, part of the monomer binds to the inner side by means of the amphiphilic units, fabricating polymer brush into nanotube lumen, which can create a soft barrier between the encapsulated guest and the external environment as a result of easing the burst effect. Hence, the specific interactions between the grafted components are sensitive to factors such as temperature and salinity, playing a key role in the mechanism of responsiveness for controlled release.

## Supplementary Materials

The following are available online at http://www.mdpi.com/2073-4360/10/2/163/s1.
