# Supplementary material for: Surface-Engineered Nanocontainers Based on Molecular Self-Assembly and Their Release of Methenamine
Source: Polymers (Basel). 2018 Feb 8;10(2):163. doi: 10.3390/polym10020163 (PMC6415060; doi:10.3390/polym10020163)
Supplement: Supplementary file 1 [file polymers-10-00163-s001.pdf]

Supporting Information

Surface-Engineered Nanocontainers Based on Molecular Self-assembly and Their Release of Cross-linking Agent

Minghui Zhang [1, 2], Jinben Wang [1, *], Pei Zhang [1, *] and Haike Yan [1]

[1] Beijing National Laboratory for Molecular Sciences, Key Laboratory of Colloid, Interface and Chemical Thermodynamics, Institute of Chemistry, Chinese Academy of Sciences, Beijing 100190, P. R. China;

zhangminghui@iccas.ac.cn; jbwang@iccas.ac.cn; zhangpei@iccas.ac.cn

[2] University of Chinese Academy of Sciences, Beijing 100049, P.R.China;

* Correspondence: jbwang@iccas.ac.cn; Tel.: +86-010-62523395

1. Experimental Section

Synthesis of amphiphilic monomer (AGC_{12})

The monomer was prepared by reaction of acryloyl chloride with BHD-C_{12}. Acryloyl chloride (2.17 g, 0.024mol) was added dropwise to an anhydrous chloroform solution of BHD-C_{12} (12.9 g, 0.02mol) in 30 mL dichloromethane solution with triethylamine (0.03mol), which was kept at 0 ºC by cooling with an external ice bath. After completely adding acryloyl chloride, the mixture was stirred, and then the temperature was allowed to rise to 35 °C stirring for 48 h. After removing the solvents by evaporation under reduced pressure, the residue was recrystallized from acetone/diethyl ether repeatedly to afford yellow solid. 1H NMR (400 MHz, CDCl3): δ:6.89−6.94 (2H, m,CH_2=CH−), 6.37-6.42 (1H, m, CH_2=CH−), 5.81-5.88 (1H, t, O−CH−), 3.64-3.69(m, 4H,-CH_2-CH-COO), 3.29-3.47(m, 4H, -CH_2 -N^+), 3.17(s, 12H,-N^+-CH_3), 2.49(t,4H,-CH_2-CH_2-), 1.80(t,4H,-CH_2-CH_3), 1.32-1.40(m,32H,-$(CH_2)_9$-CH_3), 0.88 (t,6H -CH_3).

Synthesis of grafted halloysite nanotubes

Synthesis of HNTs-COCl

The procedure for preparing acrylated halloysite nanotubes (Abbreviated as HNTs-COCl) was showed in Scheme 1, which included two processes. HNT modified with amino groups(HNT-NH_2) were synthesized using pure HNT via the following method. 10.0 g HNTs were dispersed in 60 mL of toluene with stirring for 1 h. Then, 6 mL of APTES solution was added to the solution, and the mixture was stirred for 48 h at 80 °C. The HNTs-NH_2 were filtered and washed several times with distilled water and acetone, then collected, and dried at 45°C under vacuum. HNTs-COCl was obtained via the follow method: 6.0 g dried HNTs-NH_2 powders were dispersed in 30 mL dichloromethane solution with 22ml triethylamine, and 14 ml acryloyl chloride was added dropwise to the solution, which was kept under 0 ºC by cooling with an external ice bath. After addition of acryloyl chloride, the mixture was stirred at room temperature for 3 days. The obtained product was filtered and washed several times with acetone, and the characterizations of modified HNTs were described in Figure 2A in the manuscript.

Synthesis of HNTs-AGC_{12}

1.0 g AGC_{12} monomer was dissolved into 25 mL water, in a glass reactor equipped with magnetic stirrer, thermometer, gas inlet, and water-bath thermostat. And then 2.0 g HNTs-COCl powders were added and stirred to disperse uniformly. Before polymerization, the solution was bubbled with nitrogen (N_2) for 0.5 h to remove oxygen (O_2). Then the initiators (V50, 0.05 % in the mixture) were added successively at 10 °C. The polymerization was allowed to proceed for 2 h under the atmosphere of N_2 at 40 °C. The obtained product was filtered and washed with water for 5 times to remove free polymer and monomers. Then the product were obtained by lyophilization. Then the product were obtained by lyophilization. The characterizations of modified HNTs were described in Figure 2A in the manuscript.

Synthesis of HNTs-AM-AGC_{12}

2.0 g AGC_{12} monomer and 2.0 g AM were dissolved into 50 mL water, in a glass reactor equipped with magnetic stirrer, thermometer, gas inlet, and water-bath thermostat. And then 2.0 g HNTs-COCl powders were added and stirred to disperse uniformly. Before polymerization, the solution was bubbled with nitrogen (N_2) for 0.5 h to remove oxygen (O_2). Then the initiators (V50, 0.05 % in the mixture) were added successively at 10 °C. The polymerization was allowed to proceed for 2 h under the atmosphere of N_2. The obtained products were filtered and washed with water for more than 5 times to remove free polymer and monomers. Then the product were obtained by lyophilization. The characterizations of modified HNTs were described in Figure 2A in the manuscript.

Synthesis of HNTs-53%AM-AGC_{12}

2.0 g AGC_{12} monomer and 1.06 g AM were dissolved into 50 mL water, in a glass reactor equipped with magnetic stirrer, thermometer, gas inlet, and water-bath thermostat. And then 2.0 g HNTs-COCl powders were added and stirred to disperse uniformly. Before polymerization, the solution was bubbled with nitrogen (N_2) for 0.5 h to remove oxygen (O_2). Then the initiators (V50, 0.05 % in the mixture) were added successively at 10 °C. The polymerization was allowed to proceed for 2 h under the atmosphere of N_2. The obtained products were filtered and washed with water for

more than 5 times to remove free polymer and monomers. Then the product were obtained by lyophilization. The characterizations of modified HNTs were described in Figure 2A in the manuscript.

Synthesis of HNTs-AM-53%AGC$_{12}$

1.06 g AGC$_{12}$ monomer and 2.0 g AM were dissolved into 50 mL water, in a glass reactor equipped with magnetic stirrer, thermometer, gas inlet, and water-bath thermostat. And then 2.0 g HNTs-COCl powders were added and stirred to disperse uniformly. Before polymerization, the solution was bubbled with nitrogen (N_2) for 0.5 h to remove oxygen (O_2). Then the initiators (V50, 0.05 % in the mixture) were added successively at 10 °C. The polymerization was allowed to proceed for 2 h under the atmosphere of N_2. The obtained products were filtered and washed with water for more than 5 times to remove free polymer and monomers. Then the product were obtained by lyophilization. The characterizations of modified HNTs were described in Figure 2A in the manuscript.

Synthesis of HNTs-AM

2.0 g AM monomer was dissolved into 25 mL water, in a glass reactor equipped with magnetic stirrer, thermometer, gas inlet, and water-bath thermostat. And then 2.0 g HNTs-COCl powders were added and stirred to disperse uniformly. Before polymerization, the solution was bubbled with nitrogen (N_2) for 0.5 h to remove oxygen (O_2). Then the initiators (V50, 0.05 % in the mixture) were added successively at 10 °C. The polymerization was allowed to proceed for 2 h under the atmosphere of N_2. The obtained products were filtered and washed with water for more than 5 times to remove free polymer and monomers. Then the product were obtained by lyophilization. The characterizations of modified HNTs were described in Figure 2A in the manuscript.
